# Ubenimex suppresses Pim-3 kinase expression by targeting CD13 to reverse MDR in HCC cells

**DOI:** 10.18632/oncotarget.20194

**Published:** 2017-08-10

**Authors:** Qie Guo, Zhong-Guo Sui, Wen Xu, Xiang-Hua Quan, Jia-Lin Sun, Xiao Li, Hong-Yan Ji, Fan-Bo Jing

**Affiliations:** ^1^ Department of Clinical Pharmacy, The Affiliated Hospital of Qingdao University, Qingdao, Shandong 266003, PR China

**Keywords:** Ubenimex, hepatocellular carcinoma, multi-drug resistance, CD13, Pim-3 kinase

## Abstract

Hepatocellular carcinoma (HCC) is one of the most serious cancers, with rapid progression and high mortality. However, chemotherapy of HCC is hindered by multi-drug resistance (MDR). It is urgent, therefore, to explore new approaches for overcoming MDR of HCC cells. Ubenimex, an inhibitor of CD13, has been used as an immuno-enhancer for treating hematological neoplasms and other solid tumors. Here, we demonstrate that Ubenimex can also reverse MDR in the HCC cell lines HepG2/5-FU and Bel7402/5-FU. Ubenimex inhibits the expression of the proto-oncogene, Pim-3, which is accompanied by decreased expression of BCL-2 and BCL-XL, decreased phosphorylation of Bad, and increased tumor apoptosis.

Moreover, Ubenimex decreases expression of the MDR-associated proteins P-gp, MRP3 and MRP2 to enhance intracellular accumulation of Cisplatin, for which down-regulation of Pim-3 is essential. Our results reveal a previously uncharacterized function of Ubenimex in mediating drug resistance in HCC, which suggests that Ubenimex may provide a new strategy to reverse MDR and improve HCC sensitivity to chemotherapeutic drugs via its effects on Pim-3.

## INTRODUCTION

Chemotherapy is widely used to treat cancer, often with effective results. Unfortunately, clinical practice has shown that hepatocellular carcinoma (HCC) cells are less sensitive to chemotherapeutic drugs than many other cancers due to multi-drug resistance (MDR), which is characterized by tumor cell resistance, not only to a specific drug, but also to other drugs with different structures and mechanisms [1]. Drug efflux mediated by MDR associated proteins (MRPs) of the ATP-binding cassette transporter (ABC) family, including P-gp, MRP2, and MRP3, is known to be responsible for the MDR of HCC cells [2]. Thus, certifying upstream mechanisms that regulate MDR and identifying agents that reverse MDR in HCC cells have become emergent issues.

CD13, also referred as Aminopeptidase N, is a widely expressed type II zinc-dependent metalloproteinase [3]. It promotes tumor angiogenesis, invasion, and metastasis in breast, ovarian, and anterior cancer cells by inducing enzymatic cleavage of polypeptide chains [4]. CD13 also plays a vital role in the self-renewal capacity of liver cancer stem cells (LCSCs), which are derived by the differentiation of hepatic stem cells and oval cells [5]. CD13^+^LCSCs maintain a semi-dormant state of G0/G1 phase in the hypoxic environment of the liver fibrous capsule, and the growth of new tumors is demonstrated after these cells are transplanted into the skin of immune deficient mice, but no new tumors grow after CD13 antibody is administrated [6, 7]. Moreover, CD13^+^ cells in liver cancer transplants show higher proliferation ability and significantly greater resistance to Doxorubicin and 5-fluorouracil than CD13^−^cells [8].

Recent evidence suggests that CD13 may activate the expression of ABC family proteins to induce drug resistance. *In vivo* results demonstrate that stronger lung metastasis ability, as well as greater resistance to Doxorubicin and Vincristine in CD13^+^ as compared to CD13^−^ MHCC-97L cells is due to the high expression of Breast Cancer Resistance Protein2 (BCRP/ABCG2) [9]. Furthermore, CD13^+^LCSCs were found to be resistant to Irinotecan and 5-fluorouracil, and these cells express ABCG2 at high levels [10]. On the other hand, Li-7, a unique CD13(+) HCC line that was developed by cancer stem cell differentiation in culture, has been shown to be resistant to Sorafenib due to the high expression of P-gp and MRP2 [11]. CD13 also induces abnormal activation of the Hedgehog signaling pathway, in which Patched serves as a signaling activator and GLI-Kruppel family members serve as downstream effectors [12]. Specifically, CD13 can act as a pseudo ligand of Patched to sensitize the Hedgehog signaling pathway, leading to the up-regulation of ABCG2, P-gp, MRP2 and MRP3, which are direct targets of Gli1 in the induction of drug resistance [13]. These results suggest that CD13 induces drug efflux primarily by increasing the expression of MRPs.

The chemical agent Ubenimex, which is known as a CD13 inhibitor, has been reported to function as an adjuvant in the treatment of leukemia and multiple myeloma by improving immune function [14]. In a previous study, we developed a covalent compound Bes-5FU by linking 5-fluorouracil and Ubenimex, which showed superior effect in inhibiting the growth of HCC cells [15]. Based on these findings, we speculated that Ubenimex can depress MDR in HCC cells by inhibiting CD13, and thus improve the activity of 5-fluorouracil against HCC. However, to our knowledge, there is no report on the application of Ubenimex for the treatment of HCC, much less for the reversal of MDR in HCC cells.

Given that chemotherapeutic drugs inhibit tumor growth mainly by promoting cell apoptosis, apoptosis resistance constitutes another important factor in the formation of MDR in HCC cells [16]. The Provirus integrating site Moloney murine leukemia virus (Pim) family of proto-oncogenes has been implicated in cancer progression and apoptosis regulation. Three Pim kinases (Pim-1, −2, and −3) with highly conserved serine/threonine kinase activity have been identified in this family [17, 18]. The newest member of the family, Pim-3, is aberrantly expressed in several cancers, particularly those of endoderm-derived organs, including the pancreas, colon, and stomach [19]. Data also suggests that Pim-3 inhibits apoptosis by phosphorylating and inactivating the pro-apoptotic BH3-only protein Bad to promote pancreatic and colorectal tumorigenesis [20, 21]. Recently, selective expression of Pim-3 in the liver has been reported to accelerate HCC development when induced by the hepatocarcinogen diethylnitrosamine in transgenic mice [22]. Moreover, our preliminary work showed that Pim-3 is highly expressed in HCC tissues and the mouse hepatoma cell line Hepa1-6, but not in normal hepatocytes and liver tissues. Results of *in vitro* and *in vivo* assays has shown that Pim-3 not only phosphorylates specific substrates of Bad, but also promotes expression of anti-apoptotic proteins such as B-Cell Lymphoma XL (BCL-XL) and B cell lymphoma 2 (BCL-2) [23]. Thus, it is likely that Pim-3 takes part in the formation of HCC by acting as an inhibitor of apoptosis, though there is no evidence that apoptosis resistance mediated by Pim-3 is associated with MDR of HCC cells.

In this study, we established the human MDR HCC cell lines HepG2/5-FU and Bel7402/5-FU, and assessed the effects of Ubenimex in increasing their sensitivity to different chemotherapeutic drugs. We demonstrated, for the first time, that Pim-3 is positively associated with the drug resistance of HCC cells. Furthermore, we demonstrated that CD13 induces Pim-3 expression and that Ubenimex decreases Pim-3 expression by targeting CD13. Ubenimex-dependent ablation of Pim-3 promotes Cisplatin-induced apoptosis by inhibiting the expression of BCL-2, BCL-XL and the phosphorylation of Bad, and also increases drug accumulation by decreasing the expression of MRPs. Our findings suggest that Ubenimex may provide a valuable therapeutic candidate for the treatment of chemoresistant HCC.

## RESULTS

### Establishment of human MDR HCC cell lines

**Figure 1:**
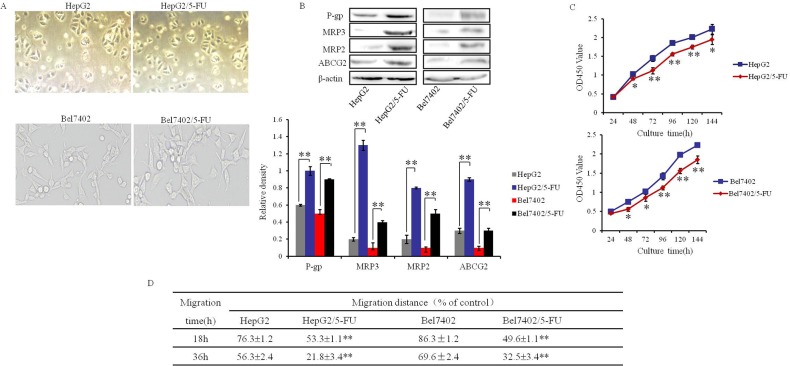
Establishment of human MDR HCC cell lines **(A)** Comparison of the morphology of HCC parental (A, left panels) and MDR cells (A, right panels). **(B)** The protein expression of P-gp, MRP2, MRP3 and ABCG2 in HCC parental and MDR cells were determined by Western blotting. β-actin was used to normalize protein loading. Representative images are shown (top panels), with means ± SD of relative densities from three independent experiments (bottom panel). **P<0.01. **(C)** The proliferative activity of HCC parental and MDR cells, represented as the OD450 value, was determined at the indicated time intervals using Cell Counting Kit-8 reagent. The proliferative activity at each time point was normalized to the activity at 0 hour. Data are shown as the means ± SD from three independent experiments. *P < 0.05 and **P < 0.01.**(D)** Migration abilities of HCC parental cells and MDR HCC cells were determined by wound scratch assay and were judged as the percentage of distance moved after 18 or 36 hours. The results are expressed as the means ± SD of three experiments. **P < 0.01.

To clarify the effect of Ubenimex on drug resistance in HCC cells, we prepared human MDR HCC cell lines HepG2/5-FU and Bel7402/5-FU by exposure of HepG2 and Bel7402 to increasing concentrations of 5-fluorouracil over four months. There was no detectable difference in morphology between the parental cells and resistant cells, which were all triangular or polygonal with uniform size and clear boundaries (Figure 1A). To assess the MDR of HepG2/5-FU and Bel7402/5-FU cells, we used the CCK-8 method to calculate the 50% inhibitory concentration (IC50) values and resistance indices (RI) after growth in 5-fluorouracil, Cisplatin and Oxaliplatin. The IC50 values were significantly increased in HepG2/5-FU and Bel7402/5-FU cells relative to their parental cells, with corresponding RIs ranging from 9.3 to 36.3 for HepG2/5-FU cells (Table 1) and 11.7 to 31.5 for Bel7402/5-FU cells (Table 2). The protein expression levels of the MRPs, including P-gp, MRP3, MRP2 and ABCG2 were also obviously higher in HepG2/5-FU and Bel-7402/5-FU cells than in parental cells (Figure 1B). Though the MDR cells showed a slower growth rate in the absence of chemotherapy drug (Figure 1C), they also showed higher motility than their parental cells did, with longer migration distances (Figure 1D). Collectively, these results demonstrate the successful establishment of MDR HCC cells.

**Table 1:** IC50 value and RIs of HepG2 and HepG2/5-FU cells treated with different chemotherapeutic agents

Drugs	HepG2 IC50(μmol/L)	HepG2/5-FU	
		IC50(μmol/L)	RI
5-fluorouracil	44.5±1.0	400.4±8.3**	9.3±1.5
Cisplatin	3.5±0.2	100.9±6.2**	36.3±4.2
Oxaliplatin	9.8±1.2	89.3±5.1**	10.1±1.9

IC50 values and RI were determined by the CCK-8 method. Data are expressed as means ± SD. **P < 0.01 versus the parental cells.

**Table 2:** IC50 values and RIs of Bel7402 and Bel7402/5-FU cells treated with different chemotherapeutic agents

Drugs	Bel7402 IC50(μmol/L)	Bel7402/5-FU	
		IC50(μmol/L)	RI
5-fluorouracil	38.3±0.7	455.2±3.1**	11.7±0.3
Cisplatin	3.9±0.8	102.1±5.2**	31.5±7.0
Oxaliplatin	8.9±0.4	118.5±2.2**	14.3±0.8

IC50 values and RI were determined by the CCK-8 method. Data are expressed as means ± SD. **P < 0.01 versus the parental cells.

### Ubenimex reverses MDR of HCC cells

**Figure 2:**
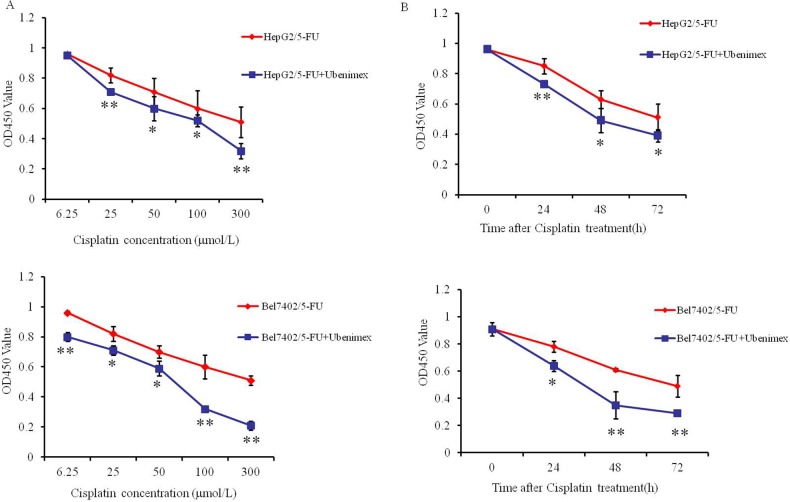
Ubenimex increases the chemosensitivity of HCC cells to Cisplatin at a range of doses and times **(A)** HepG2/5-FU (top panel) and Bel7402/5-FU (bottom panel) cells were incubated in the presence or absence of Ubenimex (400 μmol/L) for 24 h, followed by treatment with increasing concentrations of Cisplatin (6.25, 25, 50, 100, 300 μmol/L) for 48 h. **(B)** HepG2/5-FU (top panel) and Bel7402/5-FU (bottom panel) cells treated with or without Ubenimex (400 μmol/L) for 24 h were treated with Cisplatin (100 μmol/L) for 0-72 hours. Cell viability was determined by the CCK-8 method. The results are expressed as the means ± SD of three replicates. *P < 0.05 and **P < 0.01.

We next examined the effects of Ubenimex on the IC50 values and RIs in HepG2/5-FU and Bel7402/5-FU cells. Ubenimex caused a significant reduction in the IC50 values and RIs after treatment with 5-fluorouracil, Cisplatin or Oxaliplatin (Table 3 and Table 4). Moreover, Ubenimex significantly increased the sensitivity of the resistant cell lines to Cisplatin in a dose and time-dependent manner (Figure 2A and 2B). However, Ubenimex had no obvious effect on the IC50 values of various chemotherapeutic agents towards the parental HepG2 and Bel7402 cells (Supplementary Table 1). Therefore, Ubenimex may function to reverse the MDR of HCC cells that have acquired MDR.

**Table 3:** IC50 values and RIs of HepG2/5-FU cells treated with different chemotherapeutic agents in the presence or absence of Ubenimex for 24 h

Drugs	HepG2 IC50(μmol/L)	HepG2/5-FU		HepG2/5-FU+ Ubenimex	
		IC50(μmol/L)	RI	IC50(μmol/L)	RI
5-fluorouracil	44.5±1.0	405.6±7.2	9.8±1.2	176.5±5.1**	2.6±1.5**
Cisplatin	3.5±0.2	106.3±5.2	36.2±3.9	69.3±9.2**	18.9±5.6**
Oxaliplatin	9.8±1.2	85.6±4.5	9.9±1.8	53.1±8.3*	4.5±0.9*

IC50 values and RI were determined by the CCK-8 method. Data are expressed as means ± SD. **P<0.01 versus 5-fluorouracil and Cisplatin without Ubenimex; *P < 0.05 versus Oxaliplatin without Ubenimex

**Table 4:** IC50 values and RIs of Bel7402/5-FU cells treated with different chemotherapeutic agents in the presence or absence of Ubenimex for 24 h

Drugs	Bel7402 IC50(μmol/L)	Bel7402 /5-FU		Bel7402 /5-FU + Ubenimex	
		IC50(μmol/L)	RI	IC50(μmol/L)	RI
5-fluorouracil	39.2±1.2	456.2±4.1	11.4±0.4	186.2±2.5**	3.9±0.2**
Cisplatin	4.0±0.9	100.9±6.2	30.5±7.2	52.3±1.9**	11±2.9**
Oxaliplatin	8.5±0.3	120.3±3.2	14.1±0.3	60.5±1.1**	7.0±0.4**

IC50 values and RI were determined by the CCK-8 method. Data are expressed as means ±SD. **P<0.01 versus Cisplatin; 5-fluorouracil and Oxaliplatin without Ubenimex.

### Pim-3 expression is positively correlated with the MDR of HCC cells

**Figure 3:**
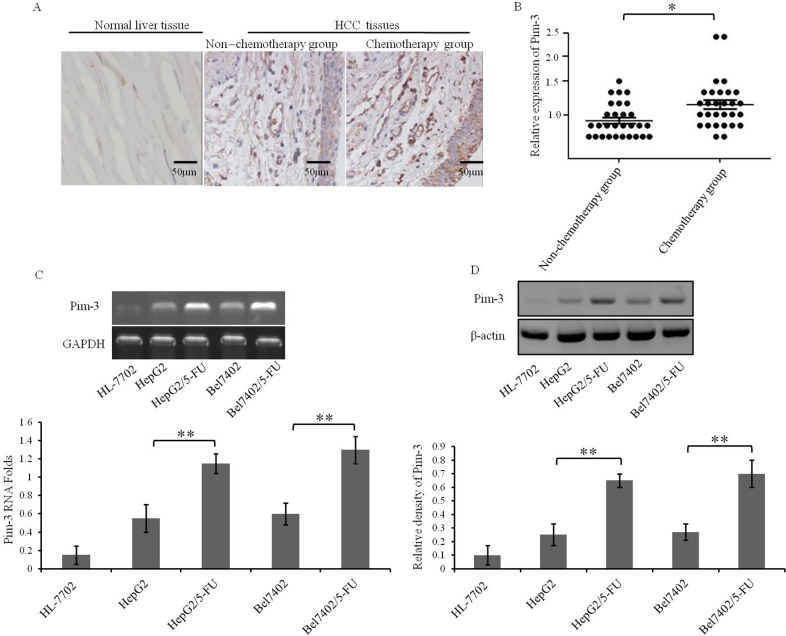
Pim-3 expression is elevated in HCC tumors from patients who underwent chemotherapy and in MDR HCC cell lines **(A)** Pim-3 expression in representative tissue sections from HCC patients with or without prior chemotherapy treatment was determined by histochemical analysis. Expression of Pim-3 appears as brown particles. **(B)** Pim-3 protein levels in the non-chemotherapy and chemotherapy groups were quantified from Western blots. The expression levels of Pim-3 were normalized to those of β-actin. The horizontal line represents the median value, and the error bars indicate the SEM. *P<0.05. **(C, D)** The expression of Pim-3 in HepG2, HepG2/5-FU, Bel7402, and Bel7402/5-FU cells were measured by Semi-quantitative reverse transcription-PCR (C) and Western blot analysis (D). The normal hepatocyte line HL-7702 was also assessed as a negative control. Data are shown as representative images (top panels) or mean ±SD relative gray values normalized to GAPDH or β-actin expression from three independent experiments (bottom panels). **P<0.01.

In a previous study, we demonstrated that a small hairpin RNA (shRNA) plasmid silencing Pim-3 expression can induce apoptosis of HCC cells and inhibit tumor proliferation [23], suggesting potential utility of Pim-3 kinase as a candidate target for HCC therapy. However, the relationship of Pim-3 with MDR in HCC cells has not been explored. Therefore, we collected 85 tissue samples from patients with primary HCC and assessed their Pim-3 expression levels. Among the HCC patients, 45 had been treated with the combination of chemotherapeutic drugs, including Doxorubicin, 5-fluorouracil and Cisplatin; 40 HCC patients had not received chemotherapy. Immunohistochemistry results demonstrated that Pim-3 was expressed at higher levels in tissue samples from the HCC patients who had received chemotherapy treatment (Figure 3A). These findings were verified by Western blotting (Figure 3B). Furthermore, semi-quantitative reverse transcription-PCR and Western blot assays demonstrated that HepG2/5-FU and Bel7402/5-FU cells have a greater abundance of Pim-3 mRNA and protein than HepG2 and Bel7402 cells (Figure 3C and 3D). These results suggest that the application of chemotherapeutic drugs increases Pim-3 expression, and that Pim-3 is also more highly expressed in MDR HCC cells than in parental cells.

To further examine the relationship between Pim-3 expression and drug resistance of HCC cells, we constructed the plasmid pTZU-Pim-3-shRNA to silence human Pim-3 mRNA and assessed its effects on drug sensitivity. Our results demonstrate that Pim-3 silencing reduces the IC50 values and RIs for HepG2/5-FU and Bel7402/5-FU cells after treatment with 5-fluorouracil, Cisplatin or Oxaliplatin (Table 5). These results suggest that Pim-3 not only promotes the occurrence of HCC, but also induces the MDR of HCC cells.

**Table 5:** IC50 values and RIs for HepG2/5-FU and Bel7402/5-FU cells after silencing of Pim-3 expression and treatment with chemotherapeutic agents for 24 h

Drugs	HepG2/5-FU		HepG2/5-FU+ pTZU-Pim-3-shRNA		Bel7402 /5-FU		Bel7402/5-FU+ pTZU-Pim-3-shRNA	
	IC50(μmol/L)	RI	IC50(μmol/L)	RI	IC50(μmol/L)	RI	IC50(μmol/L)	RI
5-fluorouracil	400.6±7.3	9.9±1.2	162.5±4.9**	2.7±1.5**	454.2±3.1	11.2±0.3	179.2±3.1**	4.2±0.4**
Cisplatin	105.3±4.2	7.4±3.9	70.2±5.1**	20.3±3.0**	101.8±4.5	27.5±6.2	49.1±2.3**	9.3±3.9**
Oxaliplatin	89.6±4.7	9.2±0.9	55.2±5.3**	6.9±0.6**	122.1±2.7	14.4±0.2	59.3±1.6**	6.5±0.3**

IC50 values and RI were determined by the CCK-8 method. Data are expressed as means±SD from three independent experiments. **P<0.01 versus 5-fluorouracil, Oxaliplatin and Cisplatin without pTZU-Pim3-shRNA transfection.

### Ubenimex targets CD13 to down-regulate Pim-3 expression of HCC cells

**Figure 4:**
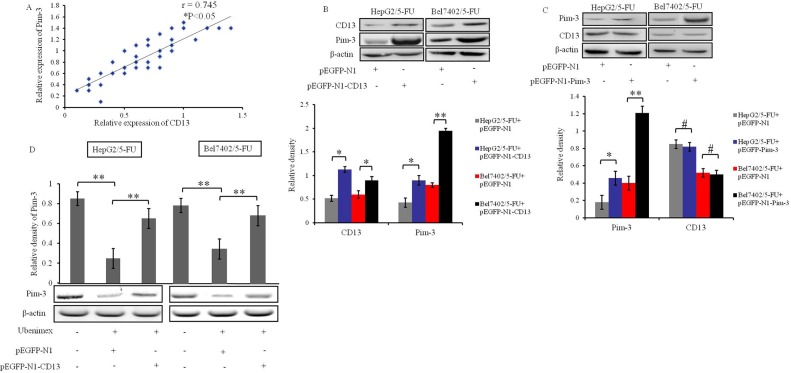
Ubenimex suppresses Pim-3 expression downstream of CD13 in HCC cells **(A)** Correlation between Pim-3 and CD13 protein level in tumor tissues from primary HCC patients after administration of chemotherapeutic drugs. β-actin protein was used as internal reference (Pearson's r = 0.745, *P < 0.05). **(B)** Effects of CD13 over-expression on Pim-3 expression in HepG2/5-FU and Bel7402/5-FU cells were assessed by Western blotting. The cells were transiently transfected with pEGFP-N1-CD13 or pEGFP-N1 plasmid for 24 h. Data are shown as representative images (top panels) and means ± SD of relative densities normalized to β-actin from three independent experiments (bottom panel). *P < 0.05 and **P < 0.01. **(C)** Effects of Pim-3 over-expression on CD13 expression in HepG2/5-FU and Bel7402/5-FU cells were determined by Western blotting. HepG2/5-FU and Bel7402/5-FU cells were transiently transfected with pEGFP-N1-Pim-3 or pEGFP-N1 plasmid for 24 h. Data are shown as representative images (top panels) and means ± SD of relative densities normalized to β-actin from three independent experiments (bottom panel). *P < 0.05, **P < 0.01 and ^#^P>0.05. **(D)** The effects of Ubenimex on Pim-3 protein levels were confirmed in HepG2/5-FU and Bel7402/5-FU cells after transfection of pEGFP-N1 or pEGFP-N1-CD13 plasmid for 24 hours. Means ± SD of relative densities normalized to β-actin from three independent experiments (D, top panels) and representative Western blotting images (D, bottom panels) are shown **P < 0.01.

As Ubenimex is known as a CD13 inhibitor [24], we considered the possibility that its ability to reverse MDR in HCC cells (Figure 2) might be explained in part by effects of CD13 on Pim-3 expression. Consistent with this possibility, expression of Pim-3 and CD13 was positively correlated in HCC tissues from the 45 HCC patients who had undergone combination chemotherapy treatment drugs (Figure 4A).

To further explore the relationship between Pim-3 and CD13 expression, we constructed CD13 and Pim-3 over-expression plasmids. Over-expression of CD13 in HepG2/5-FU and Bel7402/5-FU cells up-regulated the protein levels of Pim-3 (Figure 4B), but over-expression of Pim-3 had no effect on the expression of CD13 (Figure 4C). These results indicate that CD13 functions upstream of Pim-3 to promote its expression in HepG2/5-FUand Bel7402/5-FU cells. Interestingly, there was no significant correlation between the expression of CD13 and Pim-3 in parental HepG2 (Supplementary Figure 1) or Bel7402 cells (data not shown), which suggests that additional factors may influence CD13 up-regulation of Pim-3 in MDR cells.

To verify the role of CD13 in inducing Pim-3 in MDR HCC cells, we treated HepG2/5-FUand Bel7402/5-FU cells with Ubenimex. As expected, 24 h stimulation with Ubenimex decreased the expression of Pim-3; however, this decrease was abolished by transfection of pEGFP-N1-CD13 to over-express endogenous CD13 (Figure 4D). These results indicate that CD13 is essential for the down-regulation of Pim-3 by Ubenimex.

### Down-regulation of Pim-3 expression by ubenimex promotes cisplatin-induced apoptosis of HCC cells

**Figure 5:**
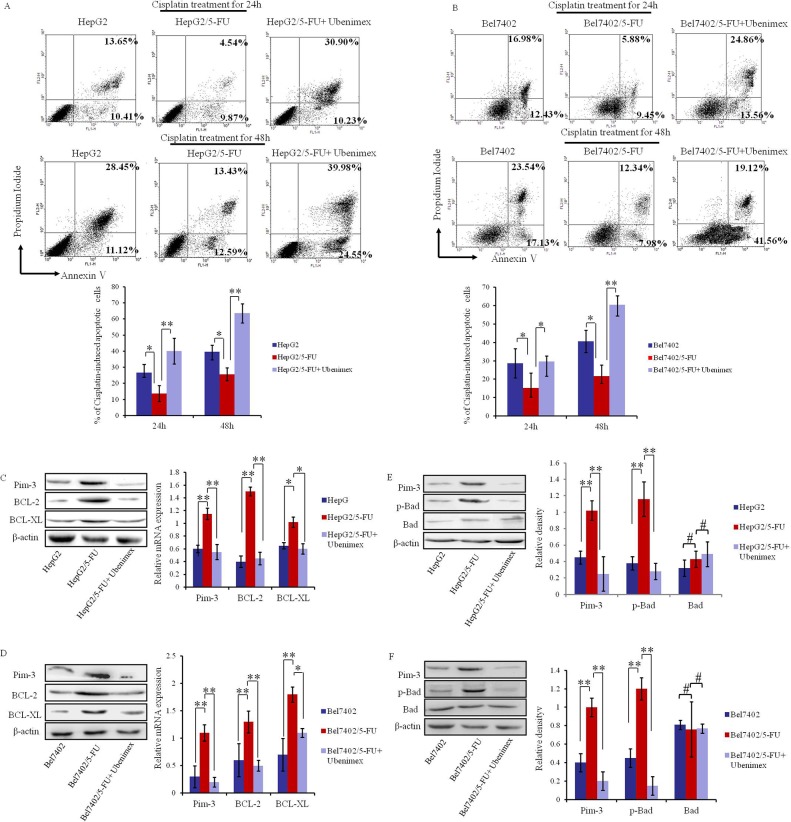
Ubenimex promotes apoptosis after Cisplatin treatment and suppresses the expression of anti-apoptotic proteins in MDR HCC cells **(A, B)** Flow cytometric analysis of Annexin V/PI double-stained HepG2/5-FU and Bel7402/5-FU cells after induction for 24 h or 48 h with Cisplatin with or without 24 h pretreatment of Ubenimex. Representative histograms are shown ((top panels), as well as the means ±SD of the proportions of apoptotic cells in three independent experiments (bottom panels). *P < 0.05 and **P < 0.01. **(C, D)** Western blotting and Real-time PCR analysis of Pim-3, BCL-XL and BCL-2 expression in HepG2 and HepG2/5-FU cells (C) or Bel7402 and Bel7402/5-FU cells (D) with or without Ubenimex treatment. Protein levels are shown as representatives (left panels) and mRNA expression is demonstrated by means ±SD of three independent experiments (right panels). *P < 0.05 and **P < 0.01. E, **(F)** Western blotting analysis of Pim-3, p-Bad and Bad in HepG2 and HepG2/5-FU cells **(E)** or Bel7402 and Bel7402/5-FU cells (F) with or without Ubenimex treatment. Representative images are shown (left panels), as well as the mean ± SD protein expression from three independent experiments (right panels). **P < 0.01 and ^#^P>0.05.

In previous studies, Pim-3 has been demonstrated to function as an important proto-oncogene that inhibits cell apoptosis by regulating the expression of apoptosis related proteins [20, 21]. Because Ubenimex suppresses Pim-3 (Figure 4D), we assessed whether Ubenimex can enhance apoptosis of MDR HCC cells after chemotherapy treatment. Annexin V/PI double staining demonstrated that Ubenimex significantly promotes apoptosis induced by 24 h or 48 h treatment of HepG2/5-FU cells with Cisplatin (Figure 5A). Similar results were obtained for Bel7402/5-FU cells (Figure 5B). However, Cisplatin-induced apoptosis in HepG2 and Bel7402 cells was not significantly affected by Ubenimex administration (Supplementary Figure 2), probably because Ubenimex can not decrease the expression of Pim-3 which was not affected by CD13 in HepG2 and Bel7402 cells (Supplementary Figure 1).

To verify these findings, we assessed the effects of Ubenimex on apoptosis-related proteins. Our results demonstrate that Ubenimex reverses the elevated expression of BCL-2 and BCL-XL in HepG2/5-FU and Bel7402/5-FU cells relative to their parental cells (Figure 5C and 5D). Furthermore, Ubenimex markedly suppressed the increased levels of phosphorylated Bad (p-Bad) in HepG2/5-FU and Bel7402/5-FUcells, but did not affect the total abundance of Bad protein (Figure 5E and 5F). Thus, the ability of Ubenimex to reverse the aberrant expression of Pim-3 and other anti-apoptotic proteins in MDR HCC cells may account for its effect in facilitating apoptosis in response to chemotherapeutic drugs.

### Reduced expression of Pim-3 by ubenimex inhibits the expression of MDR-associated proteins in HCC cells

**Figure 6:**
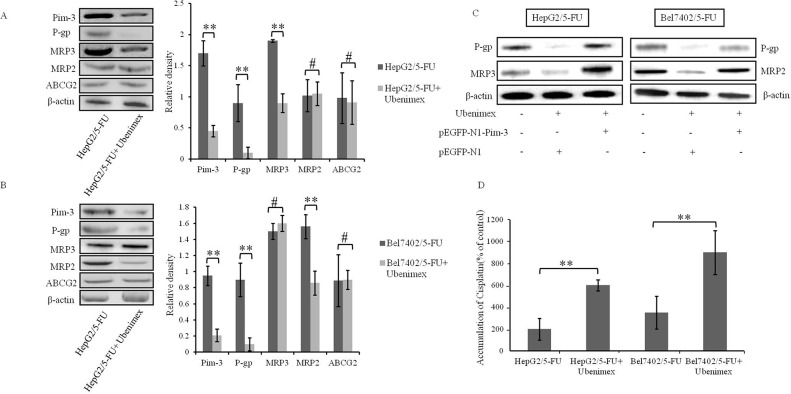
Ubenimex inhibits the expression of MRPs and enhances drug accumulation **(A, B)** The expression of Pim-3, P-gp, MRP3, MRP2 and ABCG2 in HepG2/5-FU and Bel7402/5-FU cells were determined by Western blotting analysis. Representative results (left panels) and the means ±SD (right panels) are shown. **P < 0.01 and ^#^P > 0.05. **(C)** The effects of Ubenimex on MDR-associated protein levels were confirmed in HepG2/5-FU and Bel7402/5-FU cells after transfection of pEGFP-N1 or pEGFP-N1-Pim-3 plasmid for 24 hours. Representative Western blotting images are shown. **(D)** The effect of Ubenimex (400 μmol/L) on intracellular accumulation of Cisplatin in HepG2/5-FU and Bel7402/5-FU cells. Data are expressed as means ± SD of three independent experiments. **P < 0.01.

The Pim-3 gene contains putative binding sites for STAT3 (Signal Transducer and Activator of Transcription 3), which is crucial for the phosphorylation of Bad [25]. Moreover, STAT3 activation induced by Pim-3 increases the transcription of Hif-1α, thereby up-regulating the expression of MDR1 (P-glycoprotein) gene, resulting in reduced chemosensitivity in human pancreatic cancer cells [26, 27]. To determine whether Pim-3 down-regulation by Ubenimex can also induce the expression of MRPs in HCC cells, we assessed the effects of Ubenimex on MRP expression levels. In HepG2/5-FU cells, the protein levels of P-gp and MRP3 were obviously reduced, but there was no obvious difference in MRP2 and ABCG2 expression after Ubenimex administration (Figure 6A). However, in Bel7402/5-FU cells, Ubenimex induced a remarkable reduction of P-gp and MRP2, but not MRP3 and ABCG2 (Figure 6B). More interestingly, this down-regulation of P-gp, MRP3 and MRP2 was impeded by transfection of pEGFP-N1-Pim-3 to over-express endogenous Pim-3 (Figure 6C). In contrast, Ubenimex failed to alter MRP expression in HepG2 and Bel7402 cells (Supplementary Figure 3).

Furthermore, intracellular accumulation of Cisplatin in HepG2/5-FU and Bel7402/5-FU cells was increased after Ubenimex stimulation (Figure 6D). These results suggest that Ubenimex inhibits the expression of MDR associated proteins, including P-gp, MRP3 and/or MRP2, to promote drug accumulation in MDR HCC cells, and that these effects correlate with the down-regulation of Pim-3.

## DISCUSSION

As the third leading cause of cancer-related deaths worldwide, HCC is difficult to combat due to its high degree of malignancy and poor prognosis [28]. Although chemotherapeutic drugs may prolong life expectancy, MDR often leads to chemotherapy failure in HCC. Therefore, we sought to characterize the effects of Ubenimex to elucidate molecular mechanisms that can reverse MDR in HCC and improve chemotherapy. To the best of our knowledge, this is the first study to show that (i) Ubenimex can reverse MDR of HCC cells; (ii) Pim-3 expression is positively associated with drug resistance and can be induced by CD13 in HCC cells; (iii) Ubenimex down-regulates Pim-3 expression by targeting CD13 to reverse MDR of HCC cells; (iv) Promotion of Cisplatin-induced apoptosis and inhibition of MRP expression occurs upon down-regulation of Pim-3 by Ubenimex.

At present, treatment options for patients with HCC include surgical resection, liver transplantation, molecular target therapy and chemotherapy. Surgical ablation is the most effective method for localized HCC with Child-Pugh A or B and a single tumor diameter <5 cm [29]. Liver transplantation is considered to be an effective method for the treatment of patients with end-stage liver disease or primary liver cancer, but preventing the recurrence of HCC after transplantation and improving the survival rate of patients remains a challenge [30]. In recent years, small molecule drugs targeting epidermal growth factor receptor, vascular endothelial growth factor and multiple amino acid kinase have been gradually applied to the clinical treatment of patients with HCC. However, these drugs are expensive and not widely used [31]. Therefore, chemotherapy remains critically important for patients with advanced HCC. Chemotherapeutic drugs commonly used in HCC treatment include 5-fluorouracil and its derivatives, platinums (such as Cisplatin, Carboplatin), and anthracyclines (such as Doxorubicin and Epirubicin). 5-fluorouracil combined with Adriamycin, Mitomycin and Cisplatin is the most common chemotherapy regimen used in HCC [32], while a combination of Oxaliplatin, 5-fluorouracil and Pirarubicin is also widely used in the treatment of advanced primary liver cancer, with reliable efficacy and good tolerance [33]. However, despite efforts to improve chemotherapeutic regimens, chemoresistance remains a persistent challenge.

To explore a potential new approach for overcoming chemoresistance, we constructed the human MDR HCC cell lines, HepG2/5-FU and Bel7402/5-FU. Furthermore, we confirmed that the specific CD13 inhibitor Ubenimex reverses the MDR of these cells, resulting in chemosensitivity to Cisplatin. High expression of CD13 has been shown to participate in the angiogenesis, invasion, and metastasis of tumor cells [34]. Furthermore, CD13 prevents differentiation and promotes tumorigenesis in HCC cells and LCSCs [7]. HCC cells and LCSCs with positive expression of CD13 are known to be resistant to chemotherapy due to reduced drug accumulation mediated by aberrant expression of MDR proteins [35, 36], which is consistent with our demonstrated effects of Ubenimex.

In this study, we also observed that the expression of the proto-oncogene Pim-3 is up-regulated in HCC samples from patients treated with multiple chemotherapeutic drugs. Furthermore, Pim-3 is expressed more highly in HepG2/5-FU and Bel7402/5-FU cells than in the parental cells. Previous studies suggest that Pim-3 is also highly expressed in pancreatic and gastric cancers, where it cooperates with c-Myc to promote survival [37, 38]. Furthermore, a recent study showed that Pim-3 is involved in acquired gemcitabine resistance [39]. These findings raise the possibility that Pim-3 expression may regulate MDR in HCC. Consistent with this possibility, we demonstrated that silencing of Pim-3 expression by pTZU-Pim-3-shRNA plasmid decreases the IC50 values and RI for HepG2/5-FU and Bel7402/5-FU cells to three different chemotherapeutic drugs. Thus, our results suggest that Pim-3 not only participates in the formation of HCC, but also is associated with chemoresistance.

On the basis of these findings, we also examined the association between CD13 and Pim-3 expression in HCC patient samples. Our results demonstrate that Pim-3 expression in HCC tissues of patients who underwent combined chemotherapy is positively correlated with CD13 expression. Further studies affirmed that in HepG2/5-FU and Bel7402/5-FU cells, CD13 expression induces Pim-3 expression. Consistently, Ubenimex reduced the expression of Pim-3 in drug-resistant HCC cells, but not in pEGFP-N1-CD13 cells. These findings suggest that the effects of Ubenimex on Pim-3 expression are CD13-dependent.

Apoptosis is a common pathway that mediates the anti-proliferative activity of multiple chemotherapeutic drugs, and inhibition of apoptosis is known to contribute to MDR for HCC cells in which anti-apoptotic factors, such as Nuclear factor-κB and BCL-2 are aberrantly expressed [40]. In primary liver carcinoma tissues, reactive oxygen species, which can induce Ca^2+^ influx and promote the release of cytochrome C to activate apoptosis, is refractive to the application of 5-fluorouracil [41]. High doses of Adriamycin can induce the expression of anti-apoptotic molecules BCL-2 and BCL-XL, reduce the release of Cyto-C and inhibit the apoptosis of HepG2 cells [42]. Consistently, the drug-resistant HCC cells in our study showed obvious resistance to apoptosis induced by Cisplatin, but Ubenimex resulted in a remarkable augmentation of Cisplatin-induced apoptosis. Resistance to apoptosis correlated with the high expression of BCL-2, BCL-XL and p-Bad. We previously demonstrated that the proto-oncogene Pim-3 promotes tumorigenesis of HCC by inducing anti-apoptotic proteins and impeding cell apoptosis [23]. Therefore, the reduced apoptosis in HepG2/5-FU and Bel7402/5-FU cells in the current study may result from Pim-3 overexpression, which is reversed by treatment with Ubenimex.

As a member of the proto-oncogene Pim family, Pim-3 exhibits serine/threonine kinase activity to phosphorylate multiple substrates in addition to the BCL family proteins [43]. Pim-3 has been shown to promote cell proliferation and increase gemcitabine resistance by activating AKT/β-catenin signaling [44]. Notably, the MDR1 (P-glycoprotein) gene is induced downstream of β-catenin, suggesting that Pim-3 may contribute to P-gp expression and gemcitabine resistance by up regulating β-catenin [45]. P-gp is one of several different MRP proteins that are known to contribute to the incidence of MDR in HCC [46]. MRP5 is involved in the development of intrinsic resistance, while MRP2 and MRP3 are associated with acquired resistance of HCC cells [47]. P-gp, MRP 2, MRP3 are aberrantly induced after treatment with 5-fluorouracil and Pirarubicin [48]. Furthermore, the expression of ABCG2 and P-gp is up-regulated by Adriamycin and correlates in with different stages of HCC [49]. In this study, we demonstrated that the expression of P-gp and MRP3 in HepG2/5-FU cells, P-gp and MRP2 in Bel7402/5-FU cells, were reduced significantly after treatment with Ubenimex. But the decrease of above MDR-associated proteins was abolished after Pim-3 was over-expressed in HepG2/5-FU and Bel7402/5-FU cells. It is unclear why Ubenimex reduces the expression of different MRP proteins in different cells; however, we demonstrated that for both HepG2/5-FU and Bel7402/5-FU, the end result was the intracellular accumulation of Cisplatin. In contrast, MRP levels were not affected by Ubenimex in parental HepG2 and Bel7402 cells, in which there were not distinct correlation between the expression of CD13 and Pim-3. Based on these findings, reduced expression of Pim-3 may mediate the effects of Ubenimex in inhibiting MRP expression. However, this possibility requires confirmation in future investigations.

Taken together, our results suggest that Ubenimex may serve as a potent candidate for reversing MDR of HCC cells. Furthermore, its effects may be explained, at least in part, by its ability to down-regulate the expression of Pim-3 proto-oncogene. These findings suggest a new approach toward the development of more potent cancer therapy regimens.

## MATERIALS AND METHODS

### Cell culture

Human hepatoma cell lines HepG2 and Bel7402, normal hepatocyte line HL-7702, and human ovarian cancer cell line SKOV3 were purchased from American Type Culture Collection. Peripheral blood mononuclear cells were isolated by Ficoll-Hypaque density gradient centrifugation (Amersham Pharmacia Biotech) from heparinized venous blood obtained from normal healthy volunteer donors at the Affiliated Hospital of Qingdao University (Qingdao, China). The cells were maintained in DMEM medium (GIBCO/BRL) supplemented with 10% heat-inactivated fetal bovine serum and cultured at 37°C in a humidfied atmosphere with 5% CO_2_.

### Chemicals

Ubenimex was provided by Shenzhen Main Luck Pharmaceuticals Inc. (Shenzhen, China). 5-fluorouracil was purchased from Xudong Haipu pharmaceutical Co., LTD. (Shanghai, China). Cisplatin and Oxaliplatin were obtained from Qilu Pharmaceutical Co., Ltd. (Jinan, China). Primary antibodies against human Pim-3, BCL-XL, BCL-2, Bad, and phospho-Bad were purchased from Cell Signaling Technology (Beverly, MA). Antibodies against human CD13, β-actin, P-gp, MRP2, MRP3 and ABCG2 were purchased from Santa Cruz Biotechnology (Santa Cruz, CA). Horseradish Peroxidase (HRP)-conjugated Affini-pure Goat Anti-Rabbit IgG (H+L) was acquired from Proteintech (Chicago, USA).

### Human samples

Normal liver tissue samples and tissue samples from HCC patients who had or had not undergone treatment with chemotherapeutic drugs were obtained from the Affiliated Hospital of Qingdao University (Qingdao, China), under the National Regulation of Clinical Sampling in China. The samples were immediately fresh frozen and stored at −80°C for further use in Western blotting or histochemical analysis.

### Establishment of human MDR HCC cell lines

HepG2 and Bel7402 cells in logarithmic growth phase were seeded at a concentration of 1 × 10^5^ cells/mL. After the cells were attached to the wall, culture medium containing 5-fluorouracil at initial concentrations of 2000 μg/L for HepG2 and 3000 μg/L for Bel7402 cells was added. Three days later, cell debris was discarded, and surviving cells were treated with fresh culture medium containing equivalent concentrations of 5-fluorouracil. After another three days, the cells were treated with double concentration of 5-fluorouracil. The above steps were repeated over four months, until HepG2-FU cells were able to grow stably in a concentration of 2 × 10^5^ μg/L 5-fluorouracil and Bel7402-FU cells were able to grow stably in a concentration of 3 × 10^5^ μg/L 5-fluorouracil. In the end, the resistant cell lines were maintained in Dulbecco's Modified Eagle's Medium (GIBCO/BRL) containing 5-fluorouracil at a concentration of 2 × 10^4^ μg/L (for HepG2-FU cells) or 3 × 10^4^ μg/L (for Bel7402-FU cells). Cell morphology was observed using an inverted microscope (Nikon, Japan).

### Proliferative activity and cell sensitivity assays

The proliferative activity and sensitivity of HCC cells to Cisplatin were determined using the Cell Counting Kit-8 assay (Dojindo Laboratories) according to the manufacturer's instructions. To evaluate proliferative activity, human HCC parental cell lines and MDR cells were seeded into 96-well culture plates (4 × 10^4^ cells/well) and incubated at 37°C for 0 h-144 h. To evaluate MDR cell sensitivity to Cisplatin, HepG2/5-FU and Bel7402/5-FU cells were incubated with various concentrations of Cisplatin (6.25, 25, 50, 100, 300 μmol/L) for 48 h, in the presence or absence of Ubenimex (400 μmol/L); or were incubated with Cisplatin (100 μmol/L) for 0-72 hours in the presence or absence of Ubenimex (400 μmol/L). Finally, the cells were incubated with CCK-8 (10 μL) for another 4 h, and the absorbance at 450 nm was read by a microplate reader (Bio-Rad, America). Growth curves were drawn to assess the proliferative activity or cell sensitivity.

### Wound scratch assay

HepG2/5-FU and Bel7402/5-FU cells were inoculated into 6-well culture plates for 24 h. The growth medium was replaced with serum-free medium, and the bottoms of the wells were scratched to introduce a gap. Photographs were taken at 18 and 36 h after scratching. The migration ability was represented as the percentage closure of the gap at 18 or 36 h relative to 0 h.

### Evaluation of drug resistance

The human hepatoma cell lines HepG2 and Bel7402 and the corresponding MDR cell lines in logarithmic growth were seeded into 96-well culture plates (4 × 10^4^ cells/well) and treated with or without Ubenimex (400 μmol/L) for 24 h, followed by various concentrations of 5-fluorouracil, Cisplatin or Oxaliplatin (600, 300, 100, 50, 25, 6.25 μmol/L). 48 hours later, the cells were cultured in drug-free medium (100 μL) and CCK-8 (10 μL) for another 4 h. Cell absorbance values were read at 450 nm. The inhibition rate was estimated by the following formula: Inhibition rate=(A-C) /(B-C), where A represents the absorbance value of the dosing group; B represents the absorbance value of group without dosing; and C represents the absorbance value of the blank cell group. IC50 values, also known as half maximal inhibitory concentration, were calculated by graphing the percent proliferation versus inhibitor concentration using Prism (Graphpad Software, La Jolla, CA). Resistance indices (RIs) were calculated as follows: RI = IC50 (resistant cells)/ IC50 (parental cells).

### Histochemical analysis

Tumor tissues were excised, fixed in 10% neutral buffered formalin, and embedded in paraffin for sectioning. The sections were stained with primary antibodies against Pim-3 overnight at 4°C, followed by incubation with HRP-conjugated secondary antibody for 2 hours at room temperature. The expression of Pim-3 was assessed by electron microscopy (Olympus, Japan) via granule accumulation.

### Plasmid construction

Construction of Pim-3-shRNA plasmid was performed according to previously described methods [23]. Briefly, three siRNA duplexes targeting the open reading frame of human Pim-3 were designed using BLOCK-iT RNAi Designer and were synthetized with BamHI and EcoRI overhanging ends by Sangon Biotech Co., Ltd. (Shanghai, China). Each shRNA oligonucleotide was cloned into the plasmid pTZU6+1, which contains a U6 polymerase-III (pol-III) promoter. The shRNA plasmid with most effective silencing effect were selected and named pTZU-Pim-3-shRNA.

For construction of the over-expression plasmids pEGFP-N1-CD13 and pEGFP-N1-Pim-3, we searched for the coding sequence of human CD13 and Pim-3 in Genbank, and designed PCR primers with SacI and AgeI overhangs. CD13 and Pim-3 cDNAs were amplified from peripheral blood mononuclear cells and SKOV3 cells by PCR. The PCR products were purified and recovered by gel electrophoresis, in accordance with the instructions of the Wizard® Genomic DNA Purification Kit (Promega). The annealed cDNAs were cloned into the expression plasmid pEGFP-N1, which contains SV40 and PCMV promoters. Transfection experiments using the plasmid constructs were carried out using Lipofectamine 2000 Reagent (Invitrogen) according to the manufacturer's instructions.

### Semi-quantitative reverse transcription-PCR and real-time PCR analysis

Total RNA was extracted using the E.Z.N.A.® HP Total RNA Kit (Omega Biotechnology), and cDNAs were generated using GoScript™ Reverse Transcriptase (Promega), followed by semi-quantitative reverse transcription (RT)-PCR and real-time PCR analysis. For semi-quantitative RT-PCR analysis, cDNA was amplified using pairs of primers that specifically targeting Pim-3. For real-time PCR analysis of Pim-3, BCL-XL and BCL-2, cDNAs was amplified using the SYBR®Premix Ex TaqTM Kit (Takara Biotechnology) with the ABI PRISM® 7500 Real-Time PCR System (Applied Biosystems). All PCR primers were provided by Sangon Biotech Co., Ltd. (Shanghai, China). Specific sequences were as follows: BCL-2 (forward: 5′-CGCTCTGTGGATGACTGAGT-3′, reverse:5′-GATTTGACCATTTGCCTGAAT-3′);BCL-XL (forward:5′-CCCGACCTATGATGATTCAAAAG-3′, reverse:5′-TCCCTCTCTGCTTCAGTTTC-3′);Pim-3(for- ward:5′-AAGCAGTGACCTCTACCCCTGGTGACC-3′, reverse:5′-CAAATAAATTAAACAATAAATAGCCCC-3′); GAPDH(forward:5′-GCTGGTGCTGAGTATTGCGT-3′, reverse:5′-TGGGAGGTGCTGTTGAAGTC-3′).β-actin (forward:5′-CCTAGAAGCATTTGCGGTGG-3′, reverse: 5′-GAGCTA-CGAGCTGCCTGACG). Relative gene expression was calculated in comparison to the expression of GAPDH or β-actin, and all procedures were carried out according to the manufacturer's protocol.

### Western blotting

Western blotting was performed according to a previously described method [23]. Briefly, cell or tissue lysates were prepared using a total protein extraction reagent (Proteintech). The protein samples (35 μg/lane) were separated by sodium dodecyl sulfate polyacrylamide gel electrophoresis and transferred to nitrocellulose membranes (Millipore). The membranes were blocked in Tris-buffered saline with 5% (w/v) non-fat dry milk and then incubated with primary antibodies overnight at 4°C, followed by incubation with HRP-conjugated secondary antibody for 60 minutes at room temperature. Immuno-reactive proteins were visualized using the ChemiDoc™ XRS+ System (Bio-Rad).

### Cell apoptosis analysis

Cells pre-treated with or without Ubenimex (400 μmol/L) for 24 h, were treated with Cisplatin (100 μmol/L) for 24 h or 48 h. Apoptosis analysis was carried out by staining with a human Annexin V-FITC Kit (Biolegend) according to the manufacturer's instructions on a FACS Calibur system. At least 20000 stained cells were analyzed per sample, and the percentage of cells that were Annexin V-positive were represented as the proportion of apoptotic cells.

### Cisplatin accumulation assay

HepG2/5-FU and Bel7402/5-FU cells were pre-treated with or without Ubenimex (400 μmol/L) for 24 h, and the intracellular accumulation of Cisplatin was determined using LC-MS/MS. Briefly, cells were suspended in ice-cold phosphate-buffered saline (PBS) and adjusted to 2.0 × 10^6^ cells/mL. After incubation at 37°C for 5 min, an equal volume of PBS containing Cisplatin (100 μmol/L) was added to the cell suspensions. After incubation at 37°C for an additional 30 min, the reaction was terminated by centrifugation. The cells were washed three times in ice-cold PBS and 300 μL cell lysis buffer was added (Beyotime). The intracellular accumulation of Cisplatin was determined using LC-MS/MS (Agilent Technologies, US).

### Statistical analyses

Statistical analysis was performed using the paired Student t test (two samples) and one-way ANOVA (multiple comparisons). Repeated experiments were carried out three or more times, and test results were expressed as means ± SD In all statistical analyses, P values < 0.05 were considered to indicate statistical significance. Pearson's analysis was carried out to estimate the positive correlation between Pim-3 and CD13.

## SUPPLEMENTARY MATERIALS FIGURES AND TABLE


