# Supplementary material for: Ubenimex suppresses Pim-3 kinase expression by targeting CD13 to reverse MDR in HCC cells
Source: Oncotarget. 2017 Aug 10;8(42):72652–65. doi: 10.18632/oncotarget.20194 (PMC5641159; doi:10.18632/oncotarget.20194)
Supplement: Supplementary file 1 [file oncotarget-08-72652-s001.pdf]

Ubenimex suppresses Pim-3 kinase expression by targeting CD13 to reverse MDR in HCC cells

SUPPLEMENTARY MATERIALS

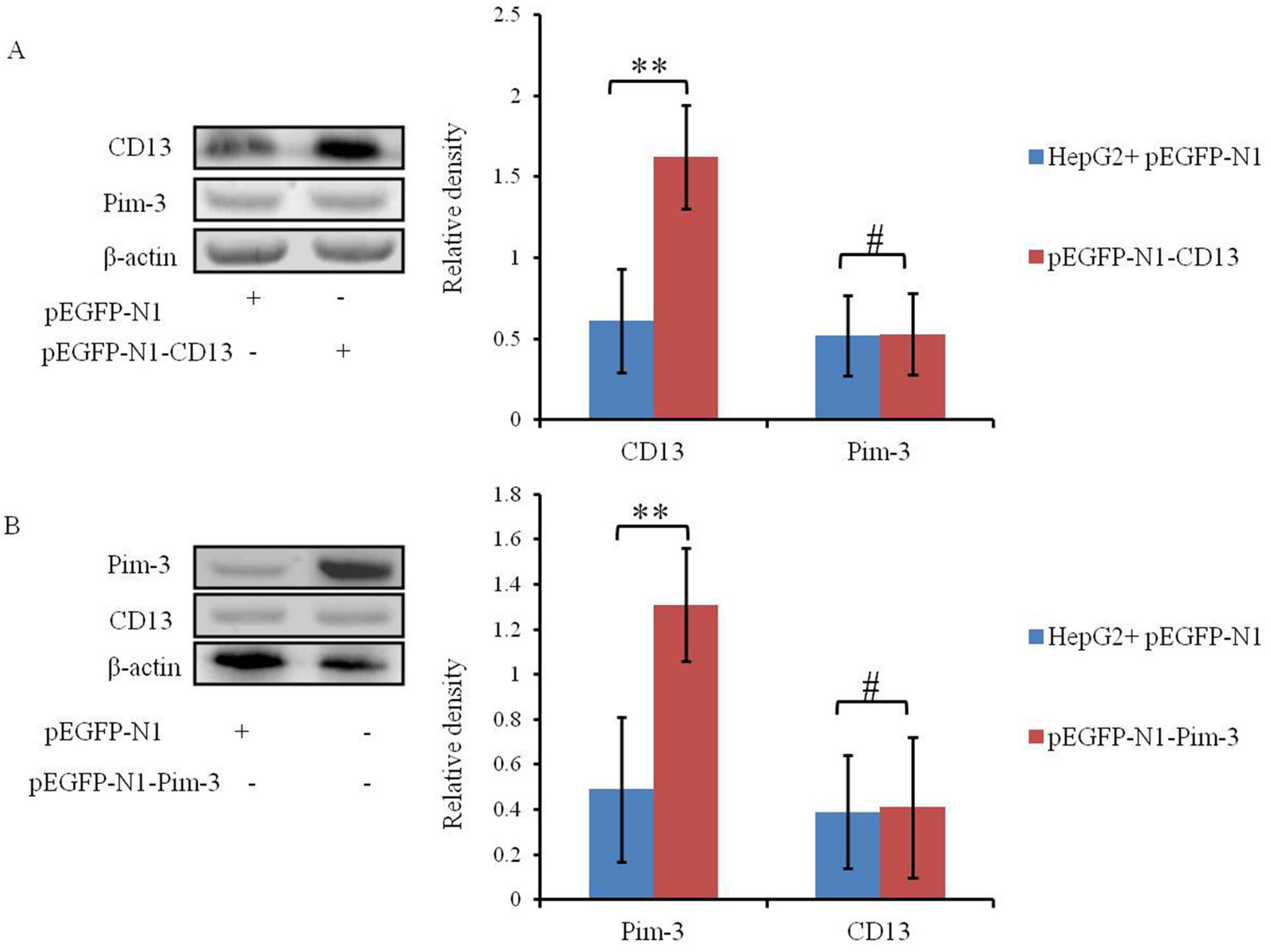

Supplementary Figure 1: CD13 does not induce Pim-3 protein expression in HepG2 cells. **(A)** Pim-3 and CD13 expression in HepG2 cells was assessed by Western blotting 24 h after transfection of pEGFP-N1-CD13 or pEGFP-N1 plasmid. **(B)** Pim-3 and CD13 expression in HepG2 cells was assessed by Western blotting 24 h after transfection of pEGFP-N1-Pim-3 or pEGFP-N1 plasmid. Data are representative (A and B, left panels) or means ± SD (A and B, right panels) from three independent experiments. **P < 0.01 and $^{\#}$P > 0.05.

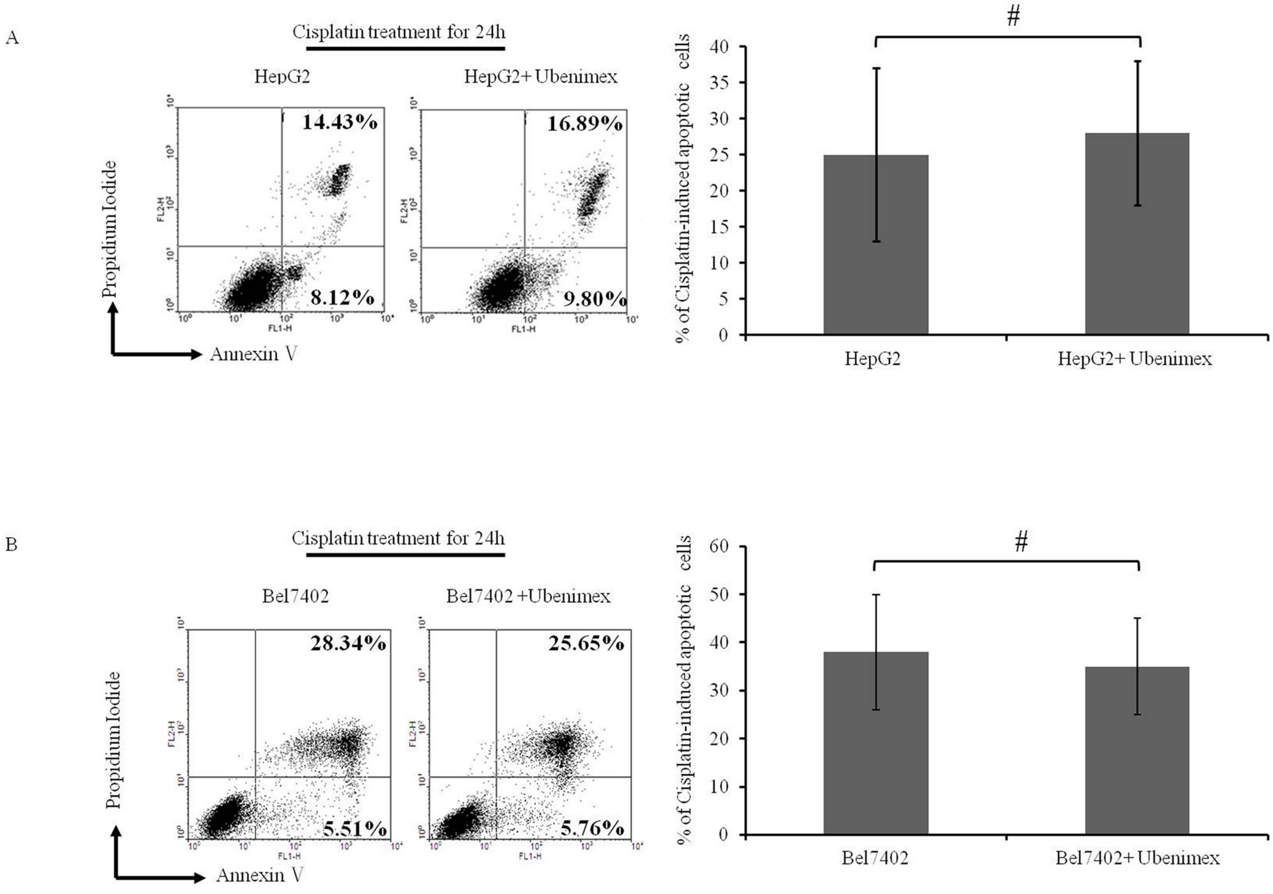

Supplementary Figure 2: Ubenimex does not stimulate Cisplatin-induced apoptosis of HepG2 and Bel7402 cells. (A, B) Apoptosis induced by Cisplatin in HepG2 (A) and Bel7402 (B) cells that were pretreated with or without Ubenimex for 24 h was detected by Annexin V/PI double staining, Data are shown as representative histograms (A and B, left panels) or means ± SD (A and B, right panels) from three independent experiments. $^{\#}$P > 0.05.

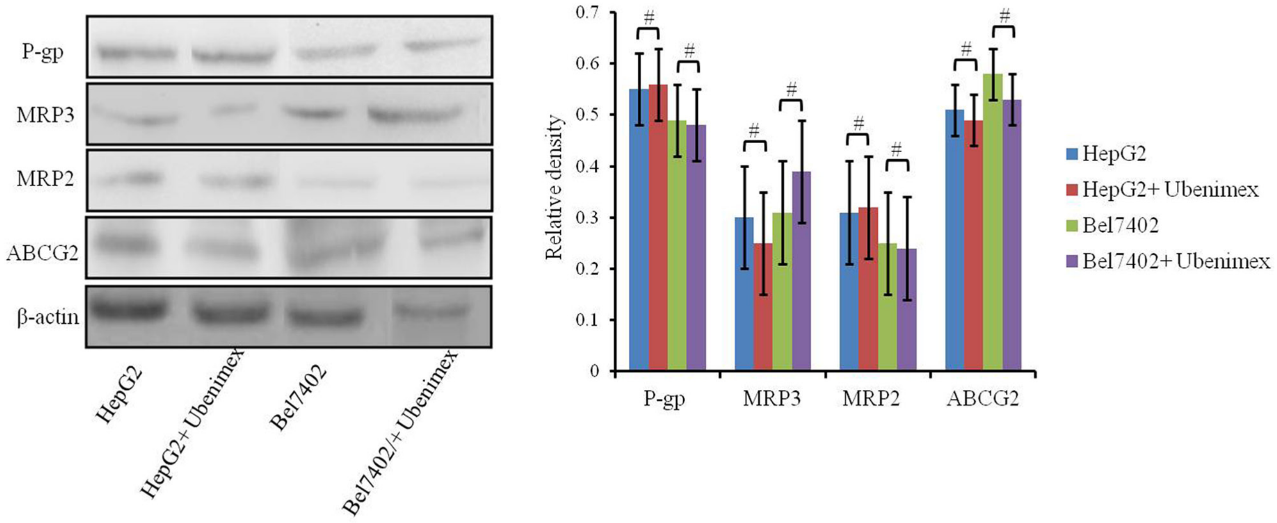

Supplementary Figure 3: Ubenimex does not alter the abundance of MRPs in HepG2 and Bel7402 cells. Protein expression of P-gp, MRP3, MRP2 and ABCG2 in HepG2 and Bel7402 cells with or without 24 h Ubenimex treatment were determined by Western blotting. Data are shown as representative images (left panels) or means ± SD (right panel) from three independent experiments. $^{\#}$P > 0.05.

Supplementary Table 1: IC50 values and RIs of HepG2 and Bel7402 cells treated with differentchemotherapeutic agents in the presence or absence of Ubenimex for 24 h

Drugs	HepG2	HepG2+ Ubenimex	Bel7402	Bel7402+Ubenimex
	IC50(μmol/L)	IC50(μmol/L)	IC50(μmol/L)	IC50(μmol/L)
5-fluorouracil	44.5±1.0	47.8±0.8#	38.3±0.7	40.9±2.1#
Cisplatin	3.5±0.2	4.6±0.1#	3.9±0.8	5.3±1.3#
Oxaliplatin	9.8±1.2	10.2±0.9#	8.9±0.4	9.0±0.2#

IC50 values and RI were determined by the CCK-8 method. Data are expressed as means±SD. #P>0.05 versus5-fluorouracil, Oxaliplatin and Cisplatin without Ubenimex.
